# Posterior reversible encephalopathy syndrome could be an underestimated variant of “reversible neurological deficits” in Systemic Lupus Erythematosus

**DOI:** 10.1186/1471-2377-12-152

**Published:** 2012-12-05

**Authors:** Bin Liu, Xuan Zhang, Feng-chun Zhang, Yuan Yao, Ri-zhi Zhou, Miao-Miao Xin, Li-Qin Wang

**Affiliations:** 1 Department of Rheumatology, The Affiliated Hospital of QingDao University Medical College, No.16 Jiang Su Lu, Shi Nan District, QingDao city, Shandong Province, 266003, China; 2 Department of Rheumatology, Peking Union Medical College Hospital, Chinese Academy of Medical Science, 1 Shuaifuyuan, Dongcheng District, Beijing 100730, China; 3 Department of Radiology, The Affiliated Hospital of QingDao University Medical College, No.16 Jiang Su Lu, Shi Nan District, QingDao city, Shandong Province, 266003, China

**Keywords:** Systemic lupus erythematosus, Neuropsychiatric lupus, Posterior reversible encephalopathy syndrome

## Abstract

**Background:**

Posterior reversible encephalopathy syndrome (PRES) has been increasingly identified in patients with systemic lupus erythematosus (SLE) owing to the advance in neuroimaging techniques. Prompt diagnosis is pivotal to improve its outcome. To analyze the clinical and radiographic profile of PRES in patients with SLE and search for the appropriate treatment strategy PRES in SLE.

**Methods:**

SLE patients who fulfilled the diagnostic criteria for PRES from August 2008 to January 2011 were evaluated at baseline, and followed to determine clinical outcomes. Data were analysis on clinical characteristics, laboratory abnormalities, treatment details, and outcomes.

**Results:**

Ten episodes of PRES in patients with SLE were identified. All patients were female, mean age of onset was 22.93 ± 2.48 years, and SLEDAI at the onset of PRES were 25.8 ± 5.7. All cases had acute onset of headache, altered mental status, stupor, vomiting, cortical blindness and seizures. Neurological symptoms were the initial manifestation of SLE in three cases. Head magnetic resonance imaging (MRI) demonstrated posterior white matter edema involving the parietal, temporal and occipital lobes, which were more conspicuous on T2 weighted spin echo and diffusion-weighted MR imaging (DWI) than on computed tomography (CT) scan. Complete clinical and radiographic recovery was observed in 8 patients after prompt treatment with corticosteroids.

**Conclusions:**

PRES might be due to lupus per se besides other traditional causative factors such as hypertension. PRES might be an underestimated variant of “reversible neurological deficits” in SLE. Prompt recognition and timely management is important to prevent permanent neurological deficits.

## Background

Systemic lupus erythematosus (SLE) is an autoimmune disease that can affect the central nervous system (CNS) [1]. Neuropsychiatric manifestations vary from severe neurological and psychiatric disorders to more subtle signs such as headache, mood disorders and defects in cognitive function [2-6]. The presentations as well as the underlying immunopathogenic mechanisms can be heterogeneous and therefore pose diagnostic and therapeutic dilemmas.

Posterior reversible encephalopathy syndrome (PRES) was first reported by Aisen et al. in 1985 [7], which was a clinical entity characterized by headache, nausea, vomiting, seizures, conscious disturbance and visual disorder with predominantly white matter abnormalities of the parieto-occipital lobes on neuroimaging [8,9]. PRES could be associated with several conditions, including hypertensive encephalopathy, eclampsia, immunosuppressive drugs and inflammatory disorders, and was reversible if treated promptly [8-12]. Literature review showed that severe hypertension (> 170/110 mmHg) and renal failure were present in the majority of previously reported cases of SLE with PRES, whereas SLE patients might also develop reversible focal deficits that respond to steroid therapy [13]. The peculiar role of SLE itself in the occurrence of PRES was not clear since PRES could be a manifestation of lupus disease activity or a consequence of immunomodulatory therapy, making the diagnosis and treatment challenging. With the development of MRI techniques, there was a high likelihood of presentation of PRES to a rheumatologist. It was important to recognize the condition early in order to minimize potential for irreversible central nervous system damage.

Here, we analyzed the clinical data of 10 cases of SLE with PRES, systematically reviewed the pathogenesis and treatment patients with SLE-associated PRES reported in the literature. The aims of the present study were to reveal the relationship between PRES and SLE, to search for the appropriate treatment strategy PRES in SLE.

## Methods

We studied the clinical data and neuroimaging of SLE patients with CNS involvement followed in department of Rheumatology of affiliated hospital Qingdao University Medical College from August 2008 to January 2011. Only SLE patients with PRES were selected, diagnosed according to the clinical and neuroradiological criteria reported in the literature. The abnormalities on imaging were defined as multiple cortico-subcortical areas of low white-matter attenuation on CT scans and as T1-weighted hypointense and T2-weighted hyperintense areas on MRI scans involving the occipital and parietal lobes bilaterally that had partially or completely resolved on follow-up scanning, when subsequent images were available. The clinical symptoms also tended to almost complete resolution after treatment [8,9]. MRI and/or CT of patients showed normal despite pronounced neuropsychiatric symptoms, and repeat MRI should be done within 2 weeks. All patients fulfilled the American College of Rheumatology (ACR) classification criteria for SLE [14], and disease activity at the time of the PRES episode was calculated using SLE Disease Activity Index (SLEDAI) [15]. Non-SLE patients, hypertensive encephalopathy, uremic encephalopathy, ischemic stroke and CNS infection were ruled out.

A brain MRI was performed with a 1.5 T GE Vectra and a standard head coil. Fast-Spin-echo T2-weighted (TR/TE/numbers of excitation = 2800/102/2), Spinecho PD-weighted (3000/40/2), Fast-Spin-echo T1-FLAIR-weighted (TR/TI/TE/numbers of excitation = 1750/720/15/2), and T2-FLAIR-weighted (TR/TI/TE/numbers of excitation = 8400/2100/125/1) sequences were performed in the axial and coronal planes, with 5 mm slice thickness. The imaging sequence for DWI was single-shot spinecho echo-planar imaging (4600/≥30 repetition time/echo time) with diffusion sensitivities b = 0 and b = 1,000 s/mm^2^. The diffusion gradients were applied sequentially in three orthogonal directions to generate three sets of axial diffusion-weighted MR imaging (DWI). Sections (5 mm thick) with 1.5 mm interslice gaps, 24 cm field of view, and 128 × 130 matrix were used for all scans. The scan time was 40 s. A composite isotropic trace image was made by multiplying the three DWI together and taking the cubic root of the result to remove the effects of diffusion anisotropy. Interpretations were made using three sets of DWI and the composite isotropic trace image. A non-contrast CT scan (Sensation 64, Siemens, Erlangen, Germany) was also available in two cases. All studies were evaluated by two expert neuroradiologists.

This study protocol was approved by the Research Ethics Committee of The Affiliation Hospital of QingDao University Medical College and followed the ethical guidelines of the 1975 Declaration of Helsinki and subsequent ones [16]. All patients gave written informed consent to participate in this study.

## Results

### Patient characteristics and clinical presentations

During the study period 732 consecutive patients were previous or newly diagnosis cases of SLE. We identified 10 patients with SLE-PRES represented 1.4% of all SLE consultations. All were female and mean age of onset was 22.93 ± 2.48 years. Three cases had PRES as a part of their initial presentation of lupus. Among 10 patients, 8 had seizures, 6 had coma, 3 had vomiting and headache, 2 had bilateral cortical blindness, and 1 had stupor. The eight patients in our series had hypertension. Before the onset of PRES, 5 had hypertension and only one patient has severe hypertension (> 170/110 mmHg). After the onset of neurologic symptoms, two patients present severity hypertension (Table 1).

**Table 1:** Clinical and laboratory characteristics

	**10 episodes, no. (%)**
Demographics	
Age at onset (years)	22.93 ± 2.48 ^*^
Female	10 (100)
SLE characteristics	
Duration (months)	20.8 ± 12.8^*^
Fever	8 (80)
Rash	8 (80)
Photosensitive	6 (60)
Ulcers	5 (50)
Arthritis/arthralgia	10 (100)
Serositis	5 (50)
Lymphadenopathy	5 (50)
Vasculitis	4 (40)
Reynaud’s phenomena	4 (40)
SLEDAI	25.8 ± 5.7^*^
Associated risk factors	
Acute hypertension	8 (80)
<160/100 mmHg	5 (50)
≥170/110 mmHg	3 (30)
Renal failure (cr. ≥1.5 mg/dl) ^b^	2 (20)
Neurological manifestations	
Seizures	8 (80)
Coma	6 (60)
Headache	3 (30)
Vomiting	3 (30)
Cortical blindness	2 (20)
Stupor	1(10)
Laboratory findings	
Leucopenia	6 (60)
Lymphopenia	2 (20)
Anemia	5 (50)
Thrombocytopenia	3 (30)
Proteinuria	8 (80)
Hypoproteinemia	8 (80)
Hypocomplementemia	8 (80)
Anti-SSA	7 (70)
Anti-SSB	2 (20)
Anti-dsDNA	6 (60)
Anti-RNP/Sm	3 (30)
Anti- Sm	2 (20)
Anti-Nucleosomes	2 (20)
aPL	4 (40)
Treatment^b^	
Methylprednisolone pulses	8 (80)
Cyclophosphamide	8 (80)
Outcome	
Complete neurological recovery	8 (80)
Residual neurological deficit	1(10)
Death	1(10)

SLEDAI: Systemic Lupus Erythematosus Disease Activity Index [23] aPL: antiphospholipid antibodies. Mean and S.D.

^b^.At clinical onset.

Patients with positive serum anti-SSA, anti-SSB, anti-dsDNA, antiphospholipid (aPL), anti-RNP/Sm, anti-Sm and anti-Nucleosome antibodies were 7/10, 2/10, 6/10, 4/10, 3/10, 2/10, 2/10, respectively. Eight patients (80%) had a history of new or previously diagnosis lupus nephritis, hypoproteinemia as well as hypocomplementemia, and 2 had renal incompetency. All patients had activated disease at onset of PRES (SLEDAI range 13–38). Lumbar puncture was carried on 8 patients, and two patients were demonstrated opening pressure beyond 180mm H_2_O. Cerebrospinal fluid examination revealed no abnormality (normal protein and glucose concentration with negative pathogenic finding).

### Neuroimaging findings

**Figure 1:**
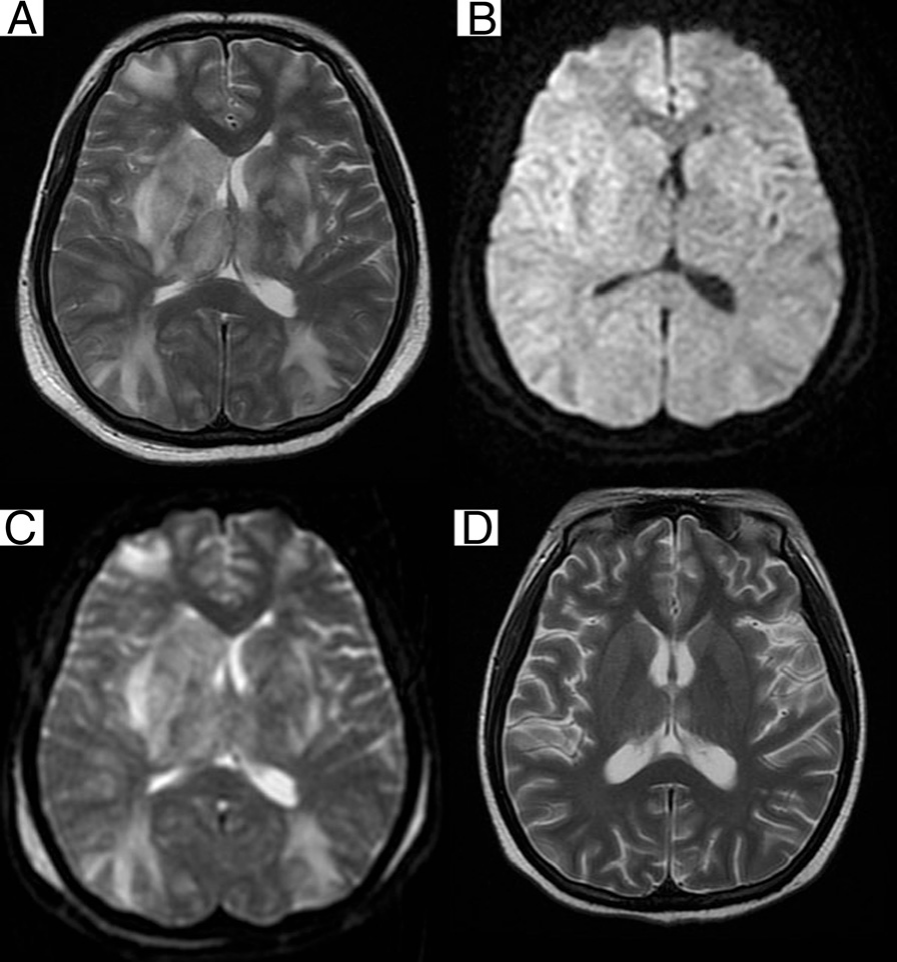
**MRI of a 24 year-old SLE patient who presented with seizure.** T2-weighted image (**A**) shows high signal lesions in deep and subcortical white matter bilaterally frontal and parietooccipital but predominantly posteriorly. Diffusion-weighted image (**B**) shows low-signal intensity area in corresponding lesion areas. Corresponding apparent diffusion coefficient (ADC) map (**C**) reveals high signal intensity area corresponding to low-signal area on diffusion-weighted image. Follow-up T2-weighted image (**D**) obtained 3 months after (A) shows complete resolution.

**Figure 2:**
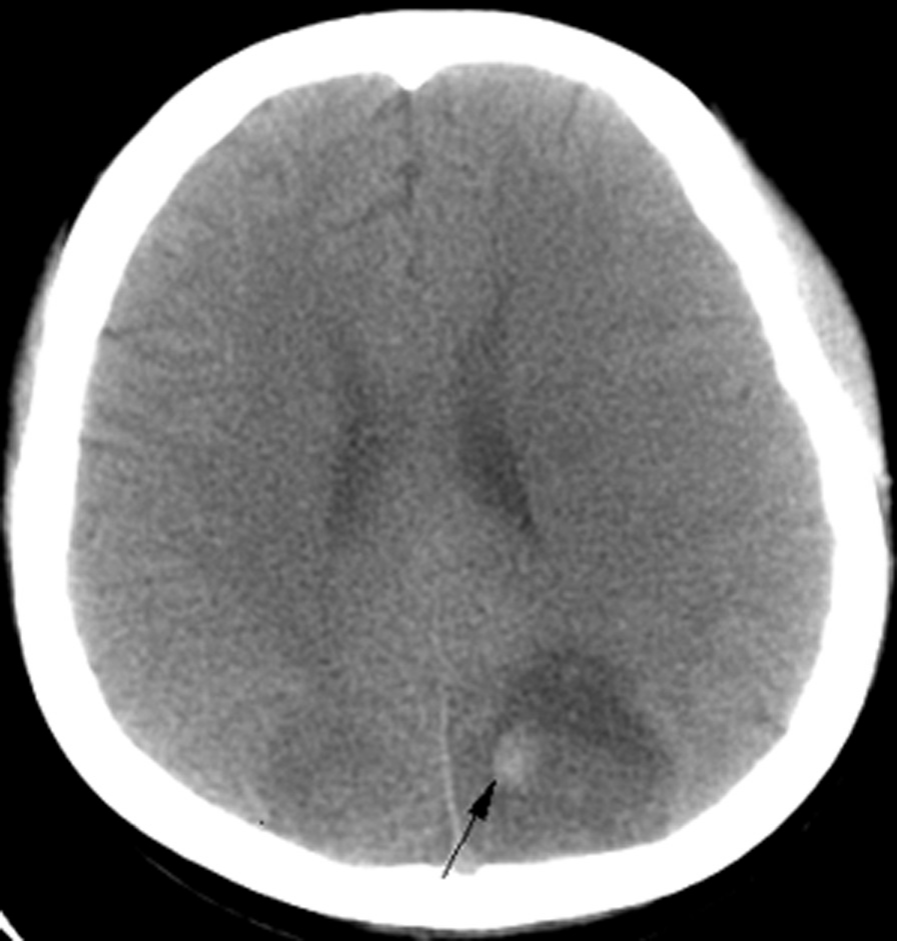
**CT of a 19 year-old SLE patient who presented with coma.** CT image on brain show showing bilateral hypodensities involving gray and white matter in posterior circulation territories as well as a right occipital hematoma (arrow).

All patients underwent cranial imaging studies; 1 underwent only CT scanning, 8 only MRI scanning, and 1 both CT and MRI scanning. Nine patients (9/10) had the initial diagnosis of PRES based on MRI findings of T2-weighted signal hyperintensity in the affected areas, while 1 patient (1/10) had the diagnosis based only on CT findings due to rapidly progressive disease. The occipital/parietal and/or cerebellar involvements were found in all 10 patients (100%), with 4 patients (40%) had abnormalities of anterior circulation. Six patients had diffused bilateral gray- and white-matter changes that were more prominent on T2-W imaging and high DWI signal intensity (Table 2) (Figure 1). One patient had a right occipital hematoma on CT (Figure 2).

**Table 2:** Imaging characteristics

	**10 episodes, no. (%)**
Edema location	
Occipital lobes	10 (100)
Unilateral	0
Bilateral	10
Parietal lobes	8 (80)
Unilateral	0
Bilateral	8
Temporal lobes	5 (50)
Unilateral	0
Bilateral	5
Frontal lobes	4(40)
Unilateral	1
Bilateral	3
Cerebellar hemispheres	2(20)
Unilateral	0
Bilateral	2
Brain stem	1(10)
Basal ganglia	1(10)
Hematoma formation	1(10)
High signal DWI	4 (40)
Cortical involvement	6 (60)

Diffusion weighted image (DWI).

**Figure 3:**
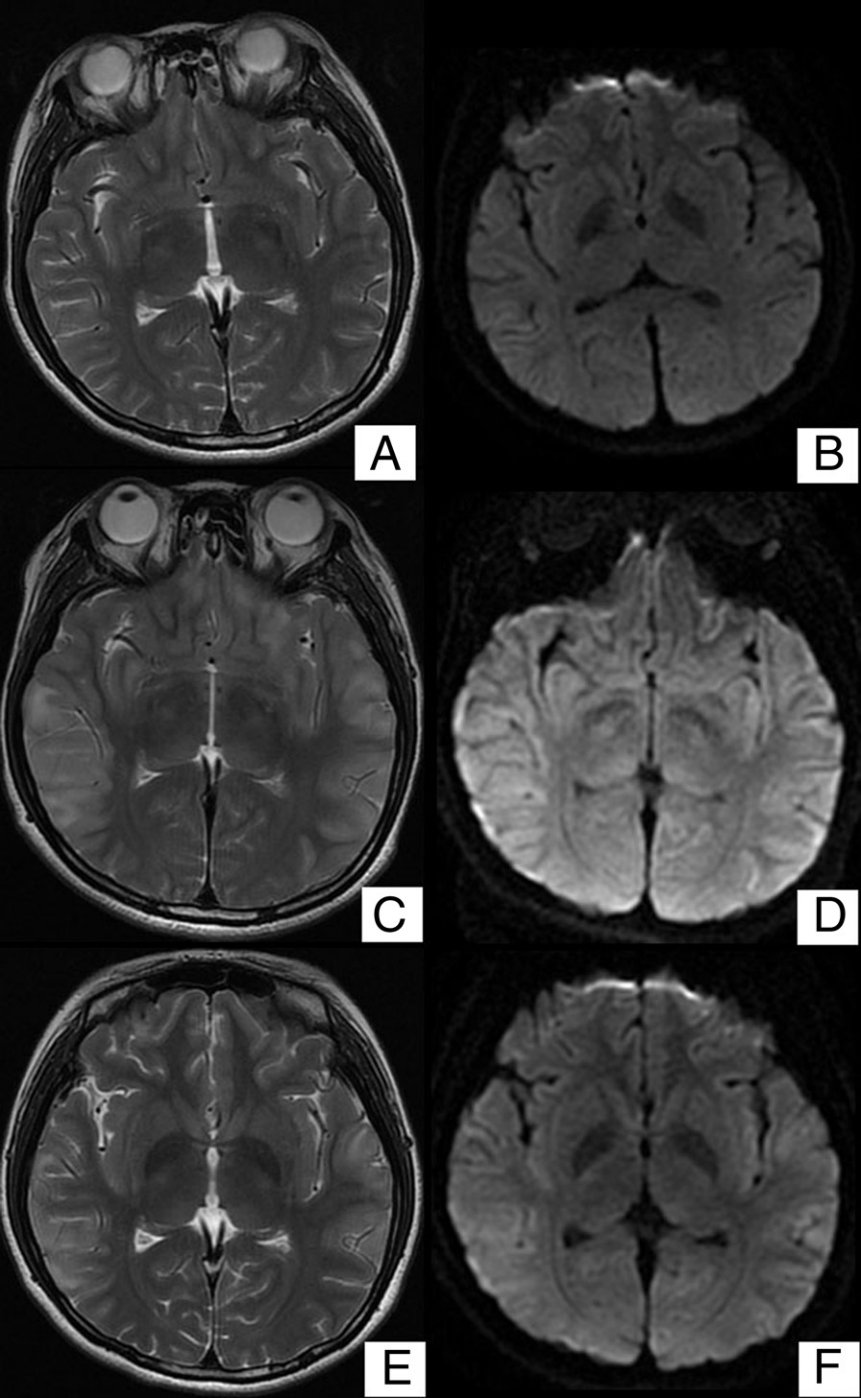
**MRI of a 19 year-old SLE patient who presented with stupor.** T2-weighted image (**A**) and Diffusion-weighted image (**B**) show normal. After 2 weeks repeat MRI, T2-weighted image (**C**) shows high signal lesions in bilaterally temporooccipital white matter. DWI (**D**) shows lesion isointensity. Follow-up T2-weighted image (**C**) obtained 2 week after (**E**) reveals almost resolution of high signal area in bilaterally lesion region. DWI (**F**) reveals lesion isointensity.

Interestingly, MRI of 2 patients showed normal despite pronounced neuropsychiatric symptoms, and a follow-up MRI suggested predominantly posterior signal abnormalities after 2 weeks (Figure 3). Follow up imaging studies were performed in 9 patients (range; from 21 to 180 days) which showed a significantly reduction in vasogenic edema in all these cases (Figure 1D).

### Treatment and outcome

Eight patients were treated with methylprednisolone pulses (1 g/day for 3 days) as well as intravenous gamma-globulin (400mg g/kg/day for 3 days) followed by oral prednisone (0.5–1.0mg/kg/day). After pulse treatment, eight patients were treated with cyclophosphamide (400mg/m^2^/week). Two patients were treated with methylprednisolone (1.0mg/kg/day) during acute phase in the department of emergency. For those patients with acute hypertension, potent antihypertensive therapy was instituted as well as general supportive measures.

Complete clinic-neruroradiographic recovery occurred in 8 patients after treatment. One patient resulted in refractory vomiting due to delayed diagnosis and therapy, notwithstanding neruroradiographic recovery at followup. One patient died of the acute episode of SLE.

## Discussion and conclusions

With advance in neuroimaging techniques, PRES has been accumulating identified over the past decade [8,17]. The similarities of the clinical manifestations between PRES, neuropsychiatric SLE (NPSLE) and lupus-related complications CNS, such as CNS infection and psychiatric conditions, often pose a diagnostic and therapeutic challenge for rheumatologist. The differential diagnosis between these entities is important, as the first requires immunosuppressive therapy, which should be avoided in the later situation [18,19].

The exact pathophysiological mechanism of PRES remains uncertain [20]. To date, three hypotheses have been proposed, which include: (i) cerebral vasoconstriction with subsequent infarcts of the brain, (ii) failure of cerebral autoregulation with consequent vasogenic oedema, and (iii) endothelial damage with disruption of the blood–brain barrier causing fluid and protein transudation in the brain [8,20-22]. The pathophysiology of PRES in SLE is also less well understood. In most cases of SLE-related PRES, immunosuppresants used to treat the SLE were suggested as causative factors, though lupus itself or SLE-related hypertension, antiphospholipid antibodies or renal failure might also be contributive. Abnormal endothelial activation, dysfunction and leukocyte tracking have recently been documented to cause brain and systemic hypoperfusion, which may be causative factors for PRES in SLE [21,23-30].

On the other hand, endothelial cell activation is one of the pathogenic hallmarks of NPSLE. It usually occurs after exposure to interleukin 1 (IL-1) and TNF-α, and may be enhanced by local release of IL-1 and IL-6 [31]. SLE patients with high SLEDAI have increased serum levels of TNF-α and other pro-inflammatory cytokines that may stimulate endothelial cells of intracranial vessels and astrocytes to produce nitric oxide (NO), causing blood–brain barrier (BBB) damage and plasma leakage [32]. In some cases the endothelial dysfunction together with hemodynamic factors may allow the escape of blood plasma and large amounts of red blood cells resulting in secondary parenchymal hematoma [27]. Histopathology showed the PRES manifestation result from NPSLE were due to focal cerebral edema associated with blood vessel injury and ischemic changes, although in many cases histopathology did not demonstrate specific lesions [28].

The occurrence of autoantibodies to SSA have been linked with vasculitis, and aPL have been involved in abnormal endothelial activation [33-35]. Our study also showed that the occurrence of anti-SSA antibodies, hypoproteinemia and hypocomplementemia might be associated with PRES. Accordingly the cause of PRES in SLE was probably due to multiple factors including lupus disease activity, hypertension, nephritis, and/or medications. Most reports had ascribed various immunosuppressive medications as causative factors. However SLE patients who were in need of immunosuppressants treatment often had active and severe disease [25,27,36], PRES could also occur in new-onset, activity SLE as well as an absolute increase in blood pressure [27]. Furthermore our series had one main distinguishing feature compared with the available literature: only 3 patients presented with severe hypertension (> 170/110 mmHg) including 2 patients had severity hypertension after the onset of PRES, compared with 95% in the reported literature. A cytotoxic effect of SLE could explain why PRES might occur in the absence of severe hypertension [11,37-39]. Consequently PRES might develop as the initial manifestation of SLE per se instead of complication of medications.

**Figure 4:**
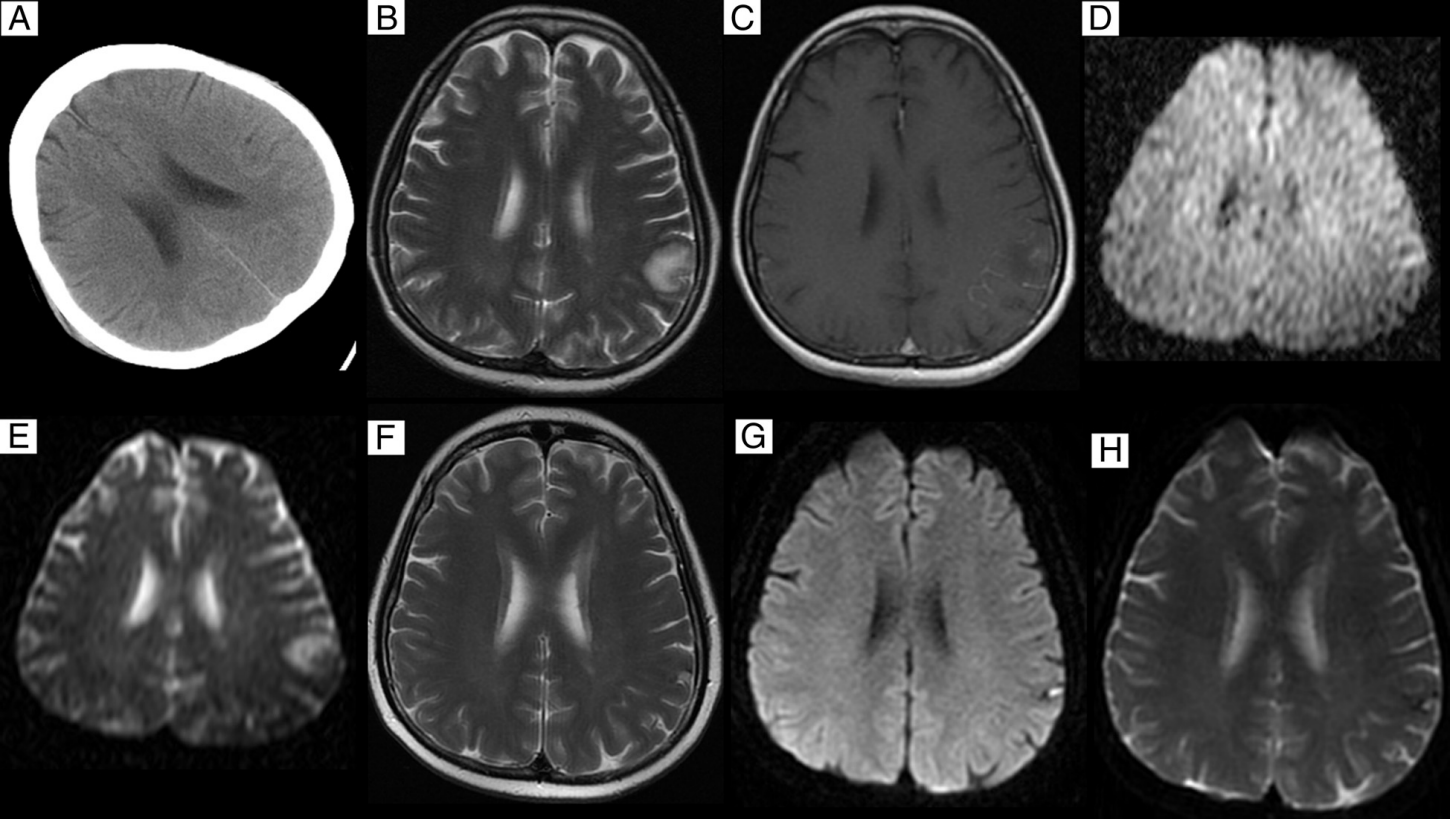
**Brain CT and MRI on admission and after treatment in a 26 year-old SLE patient who presented with seizure.** CT (**A**) shows normal but T2-weighted image (**B**) shows high signal lesions in subcortical left centroparietal white matter. On contrast-enhanced T1-weighted sequence (**C**), there is minimal enhancement at the periphery. Diffusion-weighted image (**D**) shows high-signal intensity area in corresponding lesion areas. Corresponding ADC map (**E**) reveals high signal intensity area. The abnormal lesion completely diminished in T2-weighted image (**F**) after 8 months of steroid therapy. DWI (**G**) shows high signal lesions and ADC (**H**) reveals lesion low intensity.

SLE patients might develop reversible focal neurological deficits, which responded to steroid therapy (Figure 4) [13,40-42]. On the other hand, though the subcortical and deep white matter of the posterior circulation supplied regions of the brain were usually affected in PRES, involvement of the brainstem, cerebellum, basal ganglia, frontal and temporal lobes and the cortex had also been reported in up to 56% in patients with clinical features of PRES [8,43-46]. Atypical lesion location with cortical and infratentorial involvement or involvement of its anterior circulation was not uncommon [47], which also were found in our cohort. Interesting despite pronounced neuropsychiatric symptoms, some patients had unremarkable MRI and CT scans, which were no correlate for the neurological symptoms. The possible caused for the clinico-neuroradiological discrepancies [48]. If conventional MRI findings were normal or did not provide an explanation for neuropsychiatric sign and symptoms, repeated and advanced neuroimaging might be considered. Hence, the right scan should be performed at the right time for PRES patients [49]. PRES in SLE might be an underestimated varied of “reversible neurological deficits”, which predominantly involved in posterior circulation.

The typical MRI features of PRES are diffuse hyperintensities on T2-weighted and fluid attenuated inversion recovery (FLAIR) images in the white matter in the posterior areas of the cerebral hemispheres, which spare the calcarine and paramedian occipital lobe structures and are reversible [8]. In recent years, another MR technique, echoplanar DWI findings are used in the diagnosis of PRES [50]. Regions with vasogenic edema show marked hyperintensity on apparent diffusion coefficient (ADC) and mostly iso or hypointensity on DWI [27,51]. Lack of restricted diffusion on the ADC map elaborated from DWI suggested potential reversibility. DWI can further aid in distinguishing vasogenic edema in PRES from the cytotoxic edema associated with early infracts. Cytotoxic edema has high signal intensity on DWI, due to decreased ADC, while vasogenic edema has low to intermediate signal intensity [52]. Nevertheless the lesions of some patients challenge with this knowledge, an increase in T2 signal within regions of vasogenic edema could cause slight DW1 hyperintensity [52-54]. The extent of combined T2 and DWI signal abnormalities associated with patient outcome [55]. However, whether DWI was specific in the diagnosis of patients with PRES remain to be determined [56]. Thus the exact changes detected by use of DWI in patients with PRES should be further investigation

The management of PRES in SLE is dependent on etiology. In hypertension-related and drug-induced PRES, the key to effective therapeutic includes prompt withdraw of offending agent aggressive control of blood pressure, timely anti-convulsive therapy of seizures and temporary renal support. Whereas for SLE related PRES, vigorous treatment with corticosteroids plus cyclophosphamide is directed by lupus-related major organ manifestations. Although PRES is reversible once treatment is instituted, delayed diagnosis and therapy may lead to death or irreversible neurological deficit, as also shown in our series [11,37,38]. Therefore prompt recognition and timely rational management is important to prevent permanent neurological deficits. Nevertheless controversy still exist whether immunosuppression should be used in the treatment of PRES in SLE, randomized controlled trials should be performed for addressing the strategies of therapy the situation.

In conclusion, PRES is not an exceptional condition and might be an underestimated variant of “reversible neurological deficits” in SLE. With advances in radiologic imaging, there is a high likelihood of presentation of this syndrome to a rheumatologist. Whether PRES is a presenting manifestation of SLE disease activity and of its treatment or whether it represents a neurological symptoms of SLE remain to be determined. It is important to recognize the condition early in order to ensure a very high chance of total neurological recovery in SLE patients with PRES.

## Competing interests

None of the authors have any sources of support or conflicts of interests in regards to this article.

## Authors' contributions

BL, XZ, FCZ carried out the study design, participated in the clinical studies and drafted the manuscript. BL and XZ contributed equally to this work and are both the first authors. YY, MMX carried out the data acquisition, data analysis. RZZ, LQW carried out the analysis of MRI/CT. All authors read and approved the final manuscript.

## Funding

This work was supported by National Key Technology R&D Program in the 11th Five year Plan of China (No.2008BAI59B03)

## Pre-publication history

The pre-publication history for this paper can be accessed here:

http://www.biomedcentral.com/1471-2377/12/152/prepub
